# External trigeminal nerve stimulation versus sham stimulation for attention deficit hyperactivity disorder in children and adolescents aged 7–17 years: study protocol for a pilot and feasibility randomized clinical trial

**DOI:** 10.1007/s00787-025-02786-7

**Published:** 2025-06-16

**Authors:** Henriette Edemann-Callesen, Christel-Mie Lykke Huus, Caroline Karstoft, Elín Bjarnadóttir, Aida Bikic, Pia Jeppesen, Ørjan G. Martinsen, Fred Johan Pettersen, Jane Lindschou, Sophie Juul, Maria Quistgaard, Christian Gluud, Ole Jakob Storebø

**Affiliations:** 1 Center for Evidence-Based Psychiatry, Psychiatric Research Unit, Psychiatry Region Zealand, Fælledvej 6, 4. Floor, 4200 Slagelse, Denmark; 2 Research Unit of Child and Adolescent Psychiatry in Southern Denmark, Odense, Denmark; 3 Department of Child and Adolescent Psychiatry, Psychiatry Region Zealand, 4000 Roskilde, Denmark; 4 Child and Adolescent Mental Health Services Southern Jutland, Kresten Phillipsens Vej 15, Aabenraa, Region of Southern Denmark Denmark; 5 Department of Clinical Medicine, Faculty of Health and Medical Sciences, University of Copenhagen, Copenhagen, Denmark; 6 Department of Physics, University of Oslo, Sem Sælands Vei 24, 0371 Oslo, Norway; 7 Department of Clinical and Biomedical Engineering, Oslo University Hospital, 0372 Oslo, Norway; 8 The Copenhagen Trial Unit, Centre for Clinical Intervention Research, The Capital Region, Copenhagen University Hospital ─ Rigshospitalet, Copenhagen, Denmark; 9 Mental Health Centre Stolpegaard, Copenhagen University Hospital - Mental Health Services Copenhagen, Copenhagen, Denmark; 10 Department of Psychology, University of Copenhagen, Copenhagen, Denmark; 11 Department of Regional Health Research, The Faculty of Health Sciences, University of Southern Denmark, Odense, Denmark; 12 Department of Psychology, University of Southern Denmark, 5000 Odense, Denmark

**Keywords:** Attention-deficit/hyperactive disorder, Non-invasive neuromodulation, External trigeminal nerve stimulation, Randomized feasibility, Pilot clinical trial

## Abstract

External trigeminal nerve stimulation (eTNS) is a non-invasive technique involving external cutaneous stimulation of the trigeminal nerve. In 2019, the Monarch eTNS device was approved as a treatment for children with attention-deficit/hyperactivity disorder (ADHD). The Monarch eTNS device is designed to be applied at home, which offers a certain level of convenience but also necessitates a high degree of compliance and acceptability from the families. To assess the feasibility of – and pilot a larger randomized clinical trial investigating the Monarch eTNS device versus sham for patients aged 7 to 17 years with ADHD. We will conduct a parallel-group, sham-controlled, feasibility randomised clinical trial. We will include 60 children and adolescents (aged 7 to 17 years) diagnosed with ADHD from three clinical sites in Denmark. Patients will be randomised to 4 weeks of active versus sham eTNS. Feasibility outcomes include completion of the trial; the number of eligible participants who consent to inclusion; treatment compliance; acceptability of the intervention and completion. Adverse events will be monitored throughout the trial. Exploratory clinical outcomes include ADHD core symptoms (primary) and several secondary outcomes. Autonomic functions will be evaluated by means of heart rate variability, using a heart rate sensor. This trial will allow us to evaluate the feasibility of conducting a larger randomised clinical trial investigating the use of eTNS as a home-based, non-pharmacological intervention for children and adolescents diagnosed with ADHD.

Trial registration: ClinicalTrials.gov ID: NCT06655610. Registered 23.10.2024.

## Background

Attention-deficit/hyperactivity disorder (ADHD) is a neuropsychiatric disorder often diagnosed in childhood. For a large proportion of patients, symptoms persist into adulthood, which necessitates continued treatment [1]. Central stimulants, such as methylphenidate, is recommended whenever psychosocial interventions cannot stand alone in the treatment of ADHD [2, 3]. Despite the widespread use of methylphenidate, the magnitude of the net benefits remains uncertain, and it is associated with a substantial number of adverse events [4–6].

As treatment of ADHD in most cases is initiated in childhood, interventions ought to be as non-invasive and safe as possible. In accordance, there is an increasing preference towards non-invasive approaches from both parents and patients. The use of non-invasive brain stimulation techniques seeks to target pathology-relevant brain areas, sparing other non-relevant structures [7]. External trigeminal nerve stimulation (eTNS) is such a non-invasive technique. It is based on external cutaneous stimulation of the first branch of the trigeminal nerve located in the forehead. By stimulating this nerve, it is hypothesised that afferent signals are carried from the surface of the forehead to the nucleus tractus solitarius located within the brainstem and further on to brain areas such as the prefrontal cortex, amygdala, and hippocampus [8, 9]. As such, the trigeminal nerve is potentially well-located when it comes to affecting cortical and subcortical structures involved in cognition, mood, and behaviour. In accordance, clinical investigations show that eTNS may improve symptoms in psychiatric—as well as neurological disorders including migraine [10, 11], major depressive disorder (MDD) [12, 13] and post-traumatic stress disorder (PTSD) [13]. So far, the use of eTNS has proven to be safe and well-tolerated [10–14]. The use of eTNS is furthermore approved as a treatment for drug-resistant epilepsy in the European Union, Canada, and Australia [14]. In April 2019, the FDA approved the use of eTNS as the first non-invasive medical device for the treatment of children with ADHD [15]. The approval was based on the pilot study by McGough et al. [8, 16] investigating the use of the Monarch eTNS system in 62 treatment-naive children with ADHD (age 8 to 12 years). Patients were randomly assigned to active versus sham stimulation and nightly treatment was applied by the parents at home for four weeks. Results showed a reduction of ADHD core symptoms and increase in the general function of patients receiving active eTNS compared with sham. Few mild adverse events were observed, including headache, fatigue, appetite—and sleep disturbances. No serious adverse events were reported.

The mechanism of action in ADHD remains to be fully investigated, yet it is hypothesised that eTNS may improve ADHD symptomatology by activating the fronto-basal ganglia circuit [9]. In accordance, McGough et al. [16] reported that the reduction in ADHD core symptoms following eTNS was associated with increased activation of frontal brain regions. Such change in cortical activity is similar to the mechanism thought to underlie successful pharmacological treatment for ADHD, and the localisation matches with the areas considered involved in the neuropathology of ADHD [17]. In addition, it is hypothesised that the therapeutic effect of eTNS may involve a regulation of neurotransmitters such as dopamine, and a potential induction of long-term potentiation [17, 18]. If this is in fact the case, eTNS may not only mimic the action of medicine such as methylphenidate, but also have the potential of inducing long-lasting effects within the targeted neuronal network.

The Monarch eTNS device is designed to be easily controlled by the parents at home, with stimulation being applied during night-time. This level of convenience and minimal interference of the intervention on daily activities, may be appealing to some families. In the preliminary study by Lykke Huus et al. [19], we tested the Monarch eTNS device on four children in Denmark. The study was approved by the Research Ethics Committee in Region Zealand (SJ-855). Parents generally considered the Monarch eTNS device to be user-friendly and they felt confident managing the device following the information they received prior to the study. The acceptance from the children varied, yet they were generally positive. No serious adverse events were observed.

We aim to assess the feasibility of and pilot a larger randomized clinical trial investigating the Monarch eTNS device for patients aged 7 to 17 years with ADHD in Denmark. If this Danish project shows feasibility, we will follow up with a full-sized randomised clinical trial comparable to the ongoing trials in UK [20] and US [21]. In the present trial, initial steps will be taken to explore the impact on the autonomic nervous system using heart rate variability measurements.

## Methods

### Objective

To assess the feasibility of conducting a larger randomized clinical trial investigating the Monarch eTNS device versus sham for four weeks in patients with ADHD in Denmark.

### Trial design

Parallel-group, sham-controlled, feasibility randomised clinical trial.

### Trial setting

The trial will be conducted at two outpatient child and adolescent psychiatric clinics, i.e. the Region of Southern Denmark (2 sites) and in Region Zealand (1 site).

### Staff qualifications, and training

The investigators consist of psychiatrists and psychologists with expertise in child and adolescent psychiatry, and a neuroscientist with expertise in neuromodulation techniques. All investigators will be trained to use the device properly. Dedicated investigators will be responsible for instructing the families. Software and electrical engineers with expertise in medical devices will be involved in the testing of autonomic functions.

### Ethical considerations and regulatory approval

The trial will be conducted in accordance with the requirements of the latest version of the Helsinki declaration [22] and regulation (EU) 2017/745 of the European Parliament and of the council of 5 April 2017 on medical devices (MDR) [23]. The research objective dictates use of children in the trial and thus, MDR, Article 65 is activated. Therefore, different sets of trial information will be given; a version adapted to the child’s age, a complete version to the guardians and to the patients ≥ 15 years of age. The information will be communicated in an individual meeting by a trial investigator that has experience with ADHD and children. Potential participants will be given at least 24 h to consider if they want to participate. Patients and guardians will be informed that they may withdraw their consent at any time. We will respect the child’s explicit wishes of not wanting to participate regardless of their age. Children will be informed that any concerns or questions they may have regarding the treatment, will be heard and taken seriously. As the Monarch eTNS device currently does not have an updated CE mark, an application for the approval has been sent and approved by the Danish Medicines Agency and Regional Ethics Committee for Region Zealand. The trial was registered on clinicaltrials.gov before randomisation of the first participant (ClinicalTrials.gov ID NCT06655610).

### Participants

All patients aged 7 to 17 years with a confirmed ADHD diagnosis are potential eligible for inclusion. The final eligibility will be evaluated based on the inclusion and exclusion criteria described below. The patients who have not been assessed by the Wechsler Intelligence Scale for Children (WISC-IV/V test) during the last three years will be tested with the WISC-V by psychologists from the clinics. A new WISC-V assessment will however not be conducted for children who are performing well in school [24].

### Inclusion criteria

- 7 to 17 years of age at the time of trial enrolment.
- A clinical diagnosis of ADHD according to criteria for ICD-10: F90.0, F90.1, F90.8, F98.8C [25].
  - The ADHD diagnosis must be verified by the Diagnostic and Statistical Manual for Mental Disorders (DSM-5) [26] using The Schedule for Affective Disorders and Schizophrenia for School-aged Children (K-SADS) [27].
- A total score above 24 on the ADHD rating scale (ADHD-RS) at baseline.
- Signed informed consent from parents/legal caretakers and from the patients aged ≥ 15.

We will include treatment-naïve patients, patients who previously have received stimulant medication, and patients in stable, ongoing stimulant medication (methylphenidate or dexamphetamines/lisdexamphetamine) during the time of the trial.

### Exclusion criteria

- Patients receiving atomoxetine and guanfacine at the time of trial enrolment will be excluded all together due to the impact of these medications on the arousal system, and through this the potential interference with the heart rate variability measurements.
- Epilepsy
- Electronic or metallic implants.
- Serious mental and/or somatic diseases other than ADHD, such as:
  - Pervasive developmental disorder not including Asperger’s syndrome (ICD-10 F84.0–84.4 + F84.8–84.9)
  - Schizophrenia/paranoid psychosis (ICD-10 F20-25 + F28-29)
  - Mania or bipolar disorder (ICD-10 F30 and F31)
  - Depressive psychotic disorders (ICD-10 F32.3 + F33.3)
  - Substance dependence syndrome (ICD-10 F1x.2)
  - Cardio-vascular disorders
  - Cancer
- An IQ below 70 measured by the Wechsler Intelligence Scale for Children [24, 28].
- A substantial degree of restless sleep as reported by parents or caregivers and evaluated by the physician.
- Other disabilities that may make use of Monarch problematic.

### Trial interventions

Both the active eTNS and sham stimulation will be given by the Monarch eTNS System (NeuroSigma, Inc., Los Angeles CA). Sham stimulation will serve as a control group. Stimulation will be provided by an external pulse generator, placed close to the patient’s bed and attached with thin wires to a disposable patch electrode placed on the forehead. Bilateral stimulation of the first branch of the trigeminal nerve will last for approximately eight hours nightly over the course of four weeks. Patches will be removed every morning. Power will be provided by 9 V lithium batteries, which will be replaced with a newly charged battery every day.

### Active eTNS

Active eTNS will be provided by applying single, bipolar pulses of 0.5 ms duration at a frequency of 125 Hz, with an active period of 30 s on/off. Stimulation current will range from 0.2 mili ampere (mA) to 10 mA. The level of current, which is noticeable, yet within the level of comfort, will be identified for each patient by titration at baseline. Depending on the perception of stimulation, the level of current may be altered during the four weeks of treatment, by either the guardian or adolescent in control of the settings.

### Sham stimulation

The stimulator and patches will be identical in appearance to the active treatment. The guardian/the adolescent will be informed to administer the device in the same fashion as with active treatment. The sham device will however be programmed to only apply stimulation for 30 s every hour during sleep, optimally at a frequency of maximum 2 Hz. As with the active eTNS, the sham stimulation current will range from 0.2 to 10 mA. Such settings have previously been considered to induce the sensation of a current applied to the forehead as seen with active treatment, yet without it being therapeutically effective [29, 30]. Stimulation will be directed through an internal resister, which ensures draining of batteries and need for recharging after each session. The manufacture of the eTNS device (Neurosigma) will oversee the programming the sham device.

### Records of intervention

Patients and/or their guardians are requested to keep a logbook each night. We will ask them to record the number of stimulation hours and intensity applied, whether stimulation had been interrupted and whether any issues have occurred during that night.

### Concomitant treatment

Patients in both groups will have access to one session of psychoeducation on ADHD provided by a psychologist. All families with an interest in ADHD will have access to an educational video on ADHD. Patients are allowed to continue any concomitant medications used at baseline (apart from guanfacine and atomoxetine), granted that dosages and type of medication remains stable throughout the trial. The uses of any type of concomitant interventions will be registered by the families in the logbook. Patients initiating any type of medication at the beginning of the trial will be excluded.

### Discontinuation or modification of interventions

Compliance is defined as completing the treatment in 70% of the nights during the four weeks. Abruption of treatment is only accepted if it happens less than four nights in a row. One whole night is considered of a minimum duration of five hours of uninterrupted stimulation (see feasibility outcomes below).

Families are advised to withhold from using the device in case of fever. The families are free to withdraw their informed contest at any time without providing an explanation. The trial investigators are authorised to terminate participation in case the participant is diagnosed with one of the predefined exclusion criteria or if the patients encounter intolerable adverse reactions.

#### Outcomes

### Feasibility outcomes

Feasibility outcomes will be measured at the end of the 4-week treatment period.

1. The proportion of participants assessed for eligibility who consent to inclusion and randomization
  We will compare the number of eligible participants to the number of randomized participants. We will accept a difference above 50% (95% CI 40.2% to 59.6%). A larger difference can impose difficulties with recruitment in a future randomized trial.
2. Compliance with the intervention
  We will calculate the number of participants fulfilling treatment for both the group receiving sham and active eTNS. Compliance with treatment will be defined as completing the treatment in 70% (95% CI 58.4% to 81.6%) of the nights during the four weeks. Abruption of treatment can only be accepted if it happens within less than four nights in a row.
3. Acceptability of the intervention
  The acceptability of the intervention will be assessed using a semi-structured qualitative interview guide with predefined questions:
  How did you feel about administering and controlling the device?
  How did your child feel about receiving this type of treatment?
  How was it for your child to receive home-treatment with the device in addition to other strategies provided by the clinic?
  Did the intervention have any influence on the relationship between your child and the parents/siblings/school/friends?
4. Completion of follow up
  Completion of follow-up will be defined as completing the assessment of the primary exploratory clinical outcome at the end of the intervention. The number of participants with completed outcomes will be compared to the number of participants in total. If the number of participants completing this assessment is above 90% (95% CI 82.4% to 97.6%), this will be acceptable for a future full-size trial. If the number of participants completing the assessment is below 75%, this will introduce serious problems with the interpretation of the results.
5. Use of concomitant treatment
  Any concomitant treatment or changes in medication will be assessed for each participant and subsequently evaluated for the two groups at the end of the trial.

### Safety and adverse events

Parents will be able to report directly to a dedicated investigators not involved in data analysis, with any concerns regarding potential adverse events or other safety issues during the trial. Adverse events will be measured by an adverse events rating scale at the end of treatment [29]. Height, weight and vital signs will be assessed at baseline and again following the four weeks of treatment.

### Exploratory outcomes

Exploratory clinical outcomes will be assessed at baseline and at the end of the four-week treatment period. Some specific outcomes will be assessed weekly in addition to pre – and post assessment, as indicated below. In this feasibility trial, the clinical outcomes will be used firstly in an exploratory manner.

#### Primary exploratory outcome

- ADHD core symptoms—measured by the ADHD-IV rating scale (parent- and teacher rated) [31]. Parent ratings will be assessed at baseline, weekly, and at end of treatment. Teacher ratings will be assessed at baseline and at end of treatment.
- Absence from school – measured as total hours of absence reported by logbook (parents) and through direct contact with the teachers.

#### Secondary exploratory outcomes

- Emotional liability – measured by Conners 3 Global index, Emotional liability subscale (parent – and teacher rated) [32]
- Quality adjusted life years—measured by the Child Health Utility instrument (CHU9D) (parent/self-rated) [33]
- Functional impairment – measured by the Weiss Functional Impairment Rating Scale (WFIRS) [34]
- Overall severity and improvement in symptoms – measured by the Clinical Global Impressions Scale (CGI) [35]
- Behavioural and emotional difficulties – measured by the Strengths and Difficulties Questionnaire (SDQ) (parent – and teacher rated) [36]
- Cognitive functioning—measured by the Behavior Ratings of Individual Executive Functions (BRIEF) (parent rated). Assessed at baseline. [37]
- Sleep quality—measured by The Children Sleep Habits Questionnaire (CSHQ) (parent – and teacher rated) [38]. Assessed at baseline, weekly and at end of treatment.

### Exploring the physiological impact

Autonomic functions will be assessed using heart rate variability (HRV) analysis. A chest belt capable of measuring heart rate and inter-beat interval (IBI) will be used at baseline and after the end of treatment. Measurements and calculations of analysis parameters will be done according to relevant standards and guidelines [39, 40]. Several HRV parameters can be indicative of a change in autonomous status. Since all of these are based on measured IBI, we will explore a range of parameters and compare results to existing data where such data exist.

### Participant timeline

Eligible participants will be identified by clinicians working at the respective trial sites. They will ask the families whether they are willing to be contacted by the trial researchers for further information. The trial researcher will send a letter of information and request permission to follow-up with a phone call. Following the phone call, an in-person meeting will be scheduled, in which both oral and further written information will be provided. If the families wish to participate, a second in-person meeting is scheduled. At this second meeting, informed consent is obtained as well as a standard diagnostic—and health evaluation alongside baseline assessment and HRV measurements. The child is finally included in the trial, granted they meet the inclusion criteria following the health – and diagnostic evaluation. Randomisation then commence, in which the participant is allocated to either active or sham eTNS.

They will then proceed to test the device for four weeks. Families will keep a logbook for daily reporting on how the previous night had gone by. Once a week, they will fill in an online questionnaire received by email related to the primary exploratory outcome and the reporting of any adverse events. This email will also contain questions regarding the use of the device and concomitant medication. In addition, the families will receive a weekly phone call from the trial researcher, to remind them to fill in the questionnaire and to allow the families to ask questions if needed. Families may also contact a trial researcher directly via phone throughout the trial period.

**Fig. 1:**
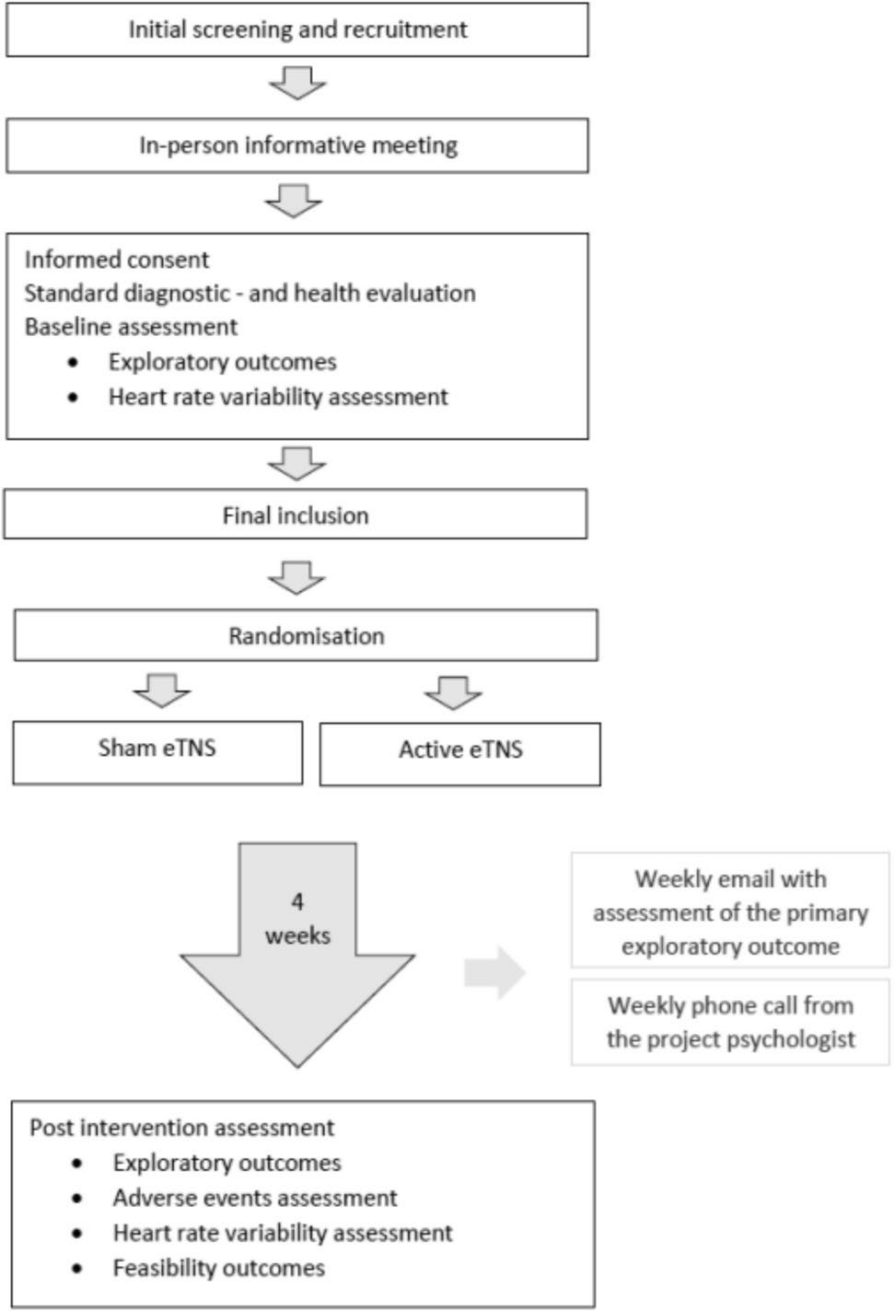
Participant timeline

Following the four weeks, an in-person meeting is scheduled, in which the families and trial investigators meet to discuss and share their experience. During this meeting, exploratory outcomes will once again be assessed, alongside HRV measurements and the rating of any adverse events. The full participant timeline is seen in Fig. 1.

### Sample size and power consideration

We will include 60 participants which corresponds to approximately 20% of the sample size needed for the full-scale randomized superiority trial. This corresponds to a power of 48% for the primary outcome (ADHD core symptoms) in this feasibility trial, indicating that the risk of type 1 error is high, and any finding thus may only be considered as exploratory.

### Randomisation and blinding

The randomization procedure will be computer-based and provided by The Copenhagen Trial Unit (CTU). The allocation sequence generation will be performed using block sizes of varying lengths concealed for the investigators. Randomization will be stratified by use of ADHD medication compared to no use of ADHD medication at entry. The allocation ratio will be 1:1. Both participants, personal and outcome assessors will be blinded to treatment allocation. Only one investigator will have access to group assignments. This investigator will also be the main person involved with any technical difficulties the families may experience during the trial. Premature disclosure of blinding may occur in case of adverse events where unblinding in considered essential for the safety and further treatment of the patient.

### Data management

Data will be collected using electronic case report forms (eCRFs) and handled using REDCap (Research Electronic Data Capture) [41, 42].

### Data analysis

Baseline characteristics will be reported and described using descriptive statistics. Continuous outcomes will be analysed by linear regression and dichotomous outcomes will be analysed using logistic regression. In the primary analysis we will include the intention-to-treat population, i.e. all randomised participants will be included in the analysis in the group to which they were randomised, even when they have received only part of the intervention. The analyses will be adjusted for the stratification variables used in the randomisation. Before the randomisation of the last participant, we will develop and publish a detailed statistical analysis plan with a detailed description of the analyses.

Statistical analyses will be performed by two blinded statisticians presenting independent reports. Our patient population includes both treatment-naïve patients as well as those already exposed to medication. In this feasibility and pilot trial, we will assess the collective effect across all patients included. We will not investigate the effect of intervention based on medication status. Such subgroup analysis will, however, be conducted in the later, larger randomised trial. A data monitoring committee will also be included in the future, larger trial.

### Quality assurance and quality control

The trial will be conducted in compliance with the protocol across all sites. Detailed instructions and Standard Operating Procedures are developed for specific tasks, as needed. The trial will be monitored by internal monitoring. Personnel from the clinical sites will be responsible for monitoring each other’s site respectively.

## Discussion

ADHD is a chronic disorder affecting children and adolescents with the core symptoms of attention deficit, hyperactivity, and impulsivity alongside a broad spectrum of co-occurring conditions [43]. The prognosis for these patients is concerning, as there is an increased risk of substance abuse, involvement in criminality and a higher mortality [44, 45]. Therefore, sufficient symptom management is needed to prevent an otherwise potential negative behavioural trajectory. Although there is some evidence supporting pharmaceutical treatment, 20% to 30% of patients do not benefit from it, and a large proportion of patients tend to withdraw from medical treatment due to adverse effects [5]. Thus, there is a need for other treatment strategies that are well-tolerated and with a high compliance rate.

Promising results have been presented for the use of the Monarch eTNS device as a treatment strategy for children with ADHD, in which a moderate clinical improvement was found in treatment naïve patients [8, 16]. However, for this type of treatment to be clinically relevant, it is essential that the opinions and perspectives of the families are assessed, including whether they indeed are interested in this type of treatment, that granted, is a different approach as opposed to the well-known use of ADHD medication. We therefore believe that conducting a feasibility trial, prior to a larger randomised trial, offers a careful and thoughtful first step, in the assessment of eTNS as a potential new treatment for ADHD within the Danish population. As such, this feasibility trial is the first trial seeking to evaluate how families perceive the use of eTNS for patients aged 7 to 17 years with ADHD in Denmark.

The Monarch eTNS system has the advantage of being a small device, with a simple interface that is easy to manage by a layperson. Treatment is controlled at home by the parents and actively applied during nighttime [16]. Given these advantages, eTNS may be appealing to patients and parents. In accordance, we expect that a high number of participants will complete the trial and that families will find this intervention acceptable. We are aware that in any trial it is difficult to meet a criterion of 100% of participants completing the trial, and thus we consider a completion rate of 90% or above as sufficient for proceeding with a larger trial. This level of completion has been chosen to accommodate a real-life setting, in which we accept that circumstances not related to the trial may arise, that necessitate termination of participation. In terms of compliance, we will consider an abruption of treatment less than four nights during the four-weeks of treatment as acceptable. This feasibility trial will also be used to determine whether the planned recruitment strategy is adequate. In our preliminary study by Lykke Huus et al. [46], we chose to solely include treatment naïve patients yet found that recruitment was hindered by the families needing immediate support. In the present feasibility trial, we expect that also including patients on stable medication may improve recruitment. By including these patients, the external validity of our trial will improve. Our preliminary study also showed that parents considered the device to be user-friendly and that they felt confident in using the device based on the information they were provided [46]. The general experience with the device, including its practical use will once again be evaluated in this trial, but now within a larger study group.

The use of non-invasive neuromodulating techniques, including eTNS, has shown to be generally well-tolerated and only induce mild adverse events [10–14, 29, 47]. In the present feasibility trial, we will extend this knowledge, by the registration of any potential adverse events, including any spontaneous adverse events reported by the patients and parents during the trial period. To investigate the preliminary effect of eTNS, this feasibility and pilot trial includes several exploratory clinical outcomes. The FDA approval of eTNS as a treatment for children with ADHD is based on the results from McGough et al. 2019, who amongst others found a reduction in ADHD core symptoms measured by ADHD-RS [16]. In the present trial, we will make use of the same outcomes employed by McGough et al. [8, 16], and the two ongoing trials in US [21] and UK [20] alongside additional measures. Due to the size of this feasibility trial, the results from these exploratory clinical outcomes are, however, to be considered preliminary and will need to be validated within a larger trial.

The application of non-invasive brain stimulation techniques, such as eTNS, aim at targeting relevant brain areas and sparing other parts of the brain, which potentially may improve treatment efficacy and reduce adverse events. The mechanism of action of eTNS is, however, largely unknown, and so far, it is mainly based on the theoretical reasoning, that stimulating the first branch of the trigeminal nerve may target pathology-relevant brain areas, as stimulation travels from the surface of the forehead to the brain stem and beyond [9]. In conjunction with investigating the feasibility of eTNS, we also wish to take the first steps in exploring the physiological impact of the device. Studies have shown that there is a correlation between activity within the autonomous nervous system and ADHD [48]. Given this correlation, and as eTNS is thought to mediate its effect via the brainstem, we wish to investigate whether eTNS potentially has an impact on the autonomous nervous system. As it is not possible to directly study the autonomous nervous system, we will have to indirectly measure organs the activity of which are affected by it. A prime candidate for this is the heart rate variability (HRV) since it is easy to measure non-invasively. For now, it is not known whether the use of eTNS therapy is modulating the autonomous system sufficiently to have an impact on HRV measurements.

### Strength and limitations

The strength of this feasibility trial includes the randomisation, the use of sham stimulation, as well as concealment of treatment allocation and blinding. We acknowledge that this feasibility trial has a small sample size. All the results from the exploratory outcomes are to be considered preliminary and need to be validated within a larger trial.

## Conclusion

This trial allows for investigating the feasibility of using the Monarch eTNS device as a non-pharmacological strategy for children and adolescents aged 7 to 17 years diagnosed with ADHD in Denmark. Results from this trial also provides the pilot for a future larger, randomised efficacy trial.

## Data Availability

No datasets were generated or analysed during the current study.
